# Vagus Nerve Stimulation Paired With Upper Extremity Rehabilitation for Chronic Stroke: Real-World Implementation and Outcomes

**DOI:** 10.1016/j.arrct.2025.100580

**Published:** 2026-01-02

**Authors:** Amanda Saylor, Laura Patrick, Chandan G Reddy, Ravi Gandhi

**Affiliations:** a Department of Sports Medicine and Rehabilitation, AdventHealth Orlando, Orlando, FL; b Orlando Neurosurgery, Orlando, FL

**Keywords:** Hemiparesis, Hemiplegial Occupational therapy, Paired VNS, Rehabilitation, Stroke, Upper extremity, Vagal nerve stimulation, Vagus nerve stimulation, Vivistim, VNS, chronic stroke

## Abstract

**Objective:**

This initiative aimed to assess real-world implementation and outcomes of a paired vagus nerve stimulation (VNS) program at AdventHealth Sports Medicine and Rehabilitation, part of the AdventHealth Central Florida system.

**Design:**

Case series of stroke patients who were implanted with a US Food and Drug Administration-approved vagus nerve stimulator and subsequently underwent at least 6 weeks of paired VNS therapy.

**Setting:**

Ten outpatient therapy clinics in central Florida.

**Participants:**

In total, 35 participants (21 men, 14 women), at least 6 months post ischemic stroke with moderate to severe upper extremity deficits. The average age was 59.2 (± SD, 13.9) years, and participants were 3.7 (± SD, 3.5) years poststroke. The mean baseline Fugl-Meyer assessment upper extremity (FMA-UE) test score averaged 30.4 (± SD, 10.2) points.

**Interventions:**

All patients were implanted with a vagus nerve stimulator and received VNS paired with outpatient occupational therapy focused on high-repetition task practice for a period of at least 6 weeks. Additionally, patients engaged in self-initiated use of VNS paired with daily tasks outside of the therapy clinic for periods of 30 minutes up to 8 times per day.

**Main Outcome Measures:**

FMA-UE test score, box and block test, and patient-specific functional scale were tested at baseline and after 6 weeks of paired VNS sessions. For program implementation, success was measured by the number of neuro-specialized occupational therapists fully trained and the number of therapy clinics in the AdventHealth Central Florida system trained and able to offer paired VNS sessions.

**Results:**

After the period of in-clinic therapy, the average FMA-UE test score improved by 10.8 (± SD, 5.4) points, with 28 of 35 (80%) participants classified as responders based on the FMA-UE test score’s minimal clinically important difference of ≥6 points. The average box and block test score change was 4.5 blocks/min (± SD, 2.9), with 10 of 23 meeting the minimal detectable change of 5.5 blocks. In just over 2 years of program implementation, 84% (21/25) of all neuro-specialized occupational therapists in AdventHealth Sports Medicine and Rehabilitation clinics are fully trained, and 10 out of 10 neuro-focused outpatient clinics are now prepared to refer and offer paired VNS sessions.

**Conclusions:**

VNS paired with rehabilitation therapy in patients with chronic ischemic stroke resulted in meaningful functional improvements aligned with individual patient goals in a real-world practice setting. Our results support findings from the pivotal VNS-REHAB (Vagus nerve stimulation paired with rehabilitation for upper limb motor function after ischaemic stroke): a randomised, blinded, pivotal, device trial and provide evidence for the feasibility of paired VNS program implementation in clinical practice.

Vagus nerve stimulation (VNS) paired with rehabilitation therapy is a US Food and Drug Administration (US FDA)-approved intervention to improve upper extremity motor function in chronic ischemic stroke patients with moderate to severe upper extremity impairment. By 6 months poststroke, two-thirds of individuals have not regained sufficient upper extremity function, as motor recovery tends to slow significantly during this chronic stage.^1^ In the pivotal, triple-blinded VNS-REHAB trial,^2^ VNS paired with task-specific practice (paired VNS) was 2-3 times more effective than intense rehabilitation therapy alone in improving both motor impairment and functional outcomes across multiple measures of arm function, including the Fugl-Meyer assessment upper extremity (FMA-UE) test and Wolf motor function test. Outcomes from these 6 weeks of paired VNS therapy sessions were sustained over 1 year.^3^ While the VNS-REHAB trial established efficacy under controlled conditions, less is known about how this therapy can be implemented and sustained in real-world health systems.

The Vivistim Paired VNS System, developed by MicroTransponder, consists of an implantable pulse generator (IPG) attached to a lead that is wrapped around the left vagus nerve in an outpatient surgical procedure. Placement of the device takes <1 hour, and patients can typically begin therapy sessions in 7-10 days. The surgical placement and technique are similar to other VNS systems used to treat refractory epilepsy and treatment-resistant depression for >25 years, with minimal risk.^4^^,^^2^^,^^5^

Stimulating the left vagus nerve enhances brain plasticity by triggering the release of neuromodulators such as norepinephrine and acetylcholine, which support learning and synaptic strengthening. In animal models, pairing VNS with active task training led to about 3 times more new connections between motor cortex neurons compared with movement training alone, thereby amplifying neuroplasticity.^6^ Together, these findings provide a scientific basis for paired VNS to enhance the effects of rehabilitation and drive greater recovery after stroke.

In the VNS-REHAB trial, intense task practice was paired with VNS for 1 group of participants (active VNS), while the other group (control VNS) also did intense task practice but did not receive active stimulations, though all were implanted with the device. All participants received approximately 90-minute sessions led by an occupational therapist (OT) or physical therapist, 3 times per week over 6 weeks (18 sessions). After 6 weeks, participants completed self-activated home-based VNS, which stimulates every 10 seconds for 30-minute periods, over the next 3 months. After 6 weeks of in-clinic therapy only, FMA-UE test scores increased by 5.0 points (± SD, 4.4) for the VNS group compared with 2.4 points (± SD, 3.8) for the control group. After 3 months of at-home use, the average total FMA-UE test score increased by 5.8 points (± SD, 6.0) from baseline compared with the 2.8 points (± SD, 5.2) for controls.

Building on the efficacy of the pivotal VNS-REHAB trial, we sought to evaluate how this therapy translates into real-world practice. This case series includes 35 patients from AdventHealth Sports Medicine and Rehabilitation, part of the AdventHealth Central Florida system, who received the Vivistim Paired VNS System implant for upper extremity impairments after chronic ischemic stroke. Here, we presented real-world experience and outcomes from patients with upper extremity impairment after chronic stroke receiving Vivistim. We also discussed the process, challenges, and success of implementing a Vivistim program across a multisite health system. To the best of our knowledge, there are no other implantable devices that need to be paired with rehabilitation therapy, so developing a program with collaboration between outpatient therapy clinics and a surgery team would be a novel undertaking for most health systems. Sharing these challenges and lessons learned may provide valuable guidance for others seeking to successfully implement a Vivistim program.

## Methods

### Participants

This prospective case series included all patients implanted at AdventHealth Central Florida between December 2022 and January 2025 who subsequently received paired VNS therapy sessions at AdventHealth Sports Medicine and Rehabilitation. During this period, 36 patients meeting the inclusion criteria were implanted with the Vivistim VNS device. All implanted patients began approximately 6 weeks of paired VNS therapy after their postoperative healing period, with therapy sessions occurring between January 2023 and March 2025. Of the 36 implanted patients, 35 completed both baseline and final assessments and were included in the analysis (21 men, 14 women). One patient was excluded because their baseline assessment had been performed at another health system and was not repeated after transfer to AdventHealth, leaving no documented baseline measurements. While the US FDA approval for paired VNS is currently limited to chronic ischemic strokes, 1 of the 35 included patients had a history of hemorrhagic stroke and received Vivistim through off-label use. Additional demographic data can be seen in table 1. Each included patient received occupational therapy sessions scheduled 3 times per week at 1 of 10 AdventHealth outpatient therapy clinics.

**Table 1:** Demographic data.

Patient Demographics				
Male		21		
Female		14		
Ischemic stroke		34		
Hemorrhagic stroke		1		

	Mean		SD	Range
Age at time of VNS implant (y)	59.2		13.9	34-84
Years poststroke	3.7		3.5	0.5-16
Baseline FMA-UE score	30.4		10.2	16-51

While it is ideal to provide the same session length and duration as the VNS-REHAB trial, because of insurance limitations, 2 patients were limited to only 1 hour of therapy each day. Rather than the typical 90-minute sessions 3 times per week, they received 60-minute sessions 4-5 times per week for 6 weeks. Though all sessions were initially scheduled within 6 weeks, illness and personal life events did change this duration for some patients and the number of sessions completed; all posttests were completed within the 14th and 20th sessions. Of note, all but 2 patients completed at least 18 sessions; 1 because of moving out of state, and the other because of scheduling conflicts and other personal obligations.

Institutional review board (IRB) approval was requested through AdventHealth’s internal review board. However, because the implementation steps and outcomes were specific to the AdventHealth organization and not broadly generalizable to all health systems or all patients with paired VNS, the IRB classified this project as a quality improvement initiative, requiring no further approval. Therefore, this project was considered IRB exempt. All patients provided informed consent.

### Patient screening and candidacy

With paired VNS being US FDA-approved in 2021, it is not yet a well-known option for stroke survivors. Potential candidates are either informed of this option by their physician, their current therapist, at a support group or other community event, or learn through their own research online. If not currently on AdventHealth Sports Medicine and Rehabilitation’s occupational therapy caseload, a physician referral for an occupational therapy evaluation was requested. Before or after the evaluation for candidacy, the MicroTransponder team provided further education to the interested patient on Vivistim Paired VNS Therapy, including how it works, what to expect, and next steps.

The OT completed a thorough evaluation of any potential candidate, including an activity of daily living interview, BBT test, and FMA-UE test, to determine whether the patient had enough active movement to pair with VNS, as well as the motivation to commit to the 6-week intensive therapy program. While there was no objective measure for motivation to engage in the therapy, the OT would ask questions such as “Are you able to come to the clinic 3 times per week?” and “Are you willing to complete exercises daily?” If deemed a therapy candidate, the patient was then scheduled for their surgical consult; if appropriate for the surgical implant procedure, insurance approval was sought. While waiting for insurance approval, 22 of 35 patients continued traditional occupational therapy services, though initial scores described in this case series were assessed after the VNS implant procedure or within 3 weeks prior.

### Paired VNS therapy

The treating OTs were trained by MicroTransponder representatives on task-specific training paired with VNS, standardized FMA-UE testing, proper use of the Stroke Application Programming Software (SAPS), and pairing of active functional movements with VNS via a hand-held trigger.

After the 7-10-day recovery period following the device implant procedure, each patient’s device was turned on in the therapy clinic by the OT. The SAPS was connected to the individual’s IPG using a wireless transmitter. Once connected, stimulation perception testing was performed beginning at 0.1 mA and ramping up in 0.1 mA increments, with the lowest sensory threshold documented. For both in-clinic and out-of-clinic use, the VNS stimulation parameters are set at 100 microseconds, 30 Hz stimulation pulses, lasting 0.5 seconds. The intensity varied slightly based on each patient’s sensory threshold and comfort level, with an initial range of 0.5-0.8 mA, though most started and remained at 0.8 mA, matching the VNS-REHAB trial. For those beginning below an intensity of 0.8 mA, the therapist, with assistance from the MicroTransponder team, would slowly increase to 0.8 mA over days to weeks as tolerated.

During therapy, stimulation was delivered through SAPS, with the therapist triggering each stimulation using a remote control during active upper extremity movement attempts. While the specific tasks varied depending on each patient’s motor function and personal goals, the emphasis remained on practicing meaningful, functional activities. For example, 1 patient’s session involved 30-80 repetitions of each task: grasping and releasing a junior-sized football, underhand tossing beanbags to a target, removing and replacing items from an overhead cabinet, opening various containers, and practicing handwriting by printing his children’s names. For simple 1-step tasks, stimulation was typically provided at a 1:1 ratio, whereas more complex multistep tasks could involve 2-3 stimulations per task completion. The primary goal of each session was to achieve 300-500 active upper extremity task repetitions, with less emphasis placed on the exact number of VNS stimulations. Nonetheless, IPG data from 21 of the 35 patients showed an average of 516 stimulations per session. For comparison, participants in the VNS-REHAB trial received about 420 stimulations per session on average.^2^

A notable difference in real-world use of paired VNS and the VNS-REHAB trial concerns the timing of when patients could start using their VNS outside of the therapy clinic. To self-activate the VNS, the patient swipes a magnet over the IPG to turn the device on for 30 minutes, providing stimulation every 10 seconds. While the VNS-REHAB trial did not include self-activated at-home stimulation until after the 6 weeks of in-clinic therapy, real-world patients can start using their VNS outside of the therapy clinic as soon as the device is turned on. Each patient was encouraged by the treating therapist to self-activate the VNS outside of therapy sessions up to 8 times per day to maximize their functional gains; with each self-activated period lasting 30 minutes, patients have the opportunity to get up to 4 hours of out-of-clinic use daily. The treating therapist provided patient-specific home exercises and functional activities, such as washing dishes or practicing typing, to be paired with the self-activated VNS outside of the clinic.

Therapists were able to download the IPG data using SAPS and review the timestamps for each self-activated VNS use, which offered an opportunity during each in-clinic session to modify the out-of-clinic program and talk through barriers to activating VNS throughout each day. Although at-home tasks were not formally documented or tracked, IPG data from 21 of the 35 patients indicated an average of 2.9 self-activations per day, corresponding to about 90 minutes of use daily during the initial 6-week period.

### Rehabilitation interventions

The primary rehabilitation intervention paired with VNS used in both the VNS-REHAB trial and with these patients was task-specific training. This method of treatment involves the use of everyday objects and the practice of ordinary everyday activities.^7^ Different from the VNS-REHAB trial, which focused therapy sessions solely on task-specific training without the use of other support interventions, the patients in this case series received a variety of other rehabilitation interventions during their 90-minute therapy sessions. For the OTs at AdventHealth Sports Medicine & Rehabilitation, these interventions are standard for poststroke patients, but may not be at all therapy sites. These adjunctive interventions were either in combination with or alternating with task-specific training. Some of these methods being discussed include functional electrical stimulation, strength training, and the use of mobile arm supports and other tools on the market. Just like the VNS-REHAB trial, the goal of each session was to achieve at least 300 active task repetitions.

Functional electrical stimulation is a method of neuromuscular electrical stimulation (NMES) during task practice shown to improve the performance of activities of daily living poststroke.^8^ Many patients with more severe distal impairments had NMES placed to facilitate digit extension, for instance, when grasping and releasing familiar items such as a cup. Typically, the same task of releasing a cup would not have been possible without the use of NMES, thus allowing for more successful task completions. While electrical stimulation, when placed on the upper extremity, is not contraindicated with VNS, other potential contraindications, such as pregnancy or having a pacemaker, were considered before using electrical stimulation in therapy sessions.^9^ Parameters and time duration varied across therapy clinics and clinicians. Some patients were instructed in the use of functional electrical stimulation at-home after instruction was given on the safe and proper use of their personal electrical stimulation device.

A 2024 systematic review concluded that combining resistance training with task-specific practice can be more effective than standard therapy for improving upper limb recovery poststroke.^10^ This finding aligns with earlier evidence showing that resistance training for the upper limb poststroke can improve motor function without increasing pain or muscle tone.^11^^,^^12^ In this case series, resistance training was often used either in isolation or in combination with task-specific training, such as supine punches with a dumbbell or adding weight to the upper extremity when practicing removing items from the fridge. Intensity was typically monitored through visible exertion or a rating of perceived exertion scale, allowing the therapist to adjust resistance appropriately.

Additionally, tools that reduce gravity or provide active assistance were used as needed. A mobile arm support, for instance, allows for gravity-reduced proximal movement of the affected upper extremity^13^ and was used in isolation or during a functional task such as folding laundry. The use of kinesiology tape was often used to facilitate digit extension^14^ to assist with item release. Typically, tape was placed distal to proximal along the digits and wrist with moderate pull of the tape to assist in grasp and release tasks. Alternatively, or in addition to, various body placements were trialed to eliminate gravity or allow gravity to assist movement. For example, hair styling could be practiced in supine for gravity-assisted external rotation. As motor function improved, a return to against-gravity tasks would be added to the therapy sessions.

### Outcome measures

The primary outcome measure used in both the VNS-REHAB trial and the present case series is the FMA-UE test score. The FMA-UE test holds good validity and reliability and is viewed as the criterion standard in assessing the function of the hemiparetic upper extremity poststroke.^15^ This 66-point test follows the Brunnstrom stages of recovery and requires the patient to move into the flexor and extensor synergies, combine synergy patterns, and move out of synergy patterns. While the VNS-REHAB trial included participants with baseline FMA-UE test scores from 20 of 66 to 50 of 66, our real-world patient selection allowed for more flexibility based on clinical judgment; thus, some patients started with scores above or below this score range. All outcome measures were assessed on the first day of in-clinic therapy postimplant or, for patients receiving occupational therapy before the implant, within the past month. They were then re-evaluated at the end of the 6 weeks by the treating OT; however, this posttesting date varied slightly but was completed on the 14th to 20th visit for all patients.

Additional outcome measures include the box and block test (BBT) and the patient-specific functional scale (PSFS). The BBT test measures gross manual dexterity and consists of a standardized box with a partition in the center. The patient is to transfer 1 block at a time from 1 side of the box (on the side of the extremity being tested) and transfer it over the partition to the opposite side; the number of blocks transferred in 60 seconds is the score, and the test should be completed for each upper extremity. While a minimally clinically important difference (MCID) has not been determined, the minimal detectable change for acute and chronic stroke patients is 5.5 points.^16^

The PSFS is a patient-focused rating scale of 3 or more functional tasks, typically determined by the patient, with a performance rating from “0” (unable to perform) to “10” (able to perform at previous level). Each task rating, from 0 to 10, is then averaged to determine the overall score on the PSFS. The functional tasks chosen by the patients also helped the OTs in planning salient treatment sessions that work toward these most valued activities of daily living. Some of the self-selected tasks included bathing independently using both arms, preparing meals, handwriting, opening containers/packages, and tying shoelaces using both hands. While there is no MCID specific to the chronic stroke population, the MCID score for musculoskeletal impairments is a change of 1.2 points.^17^

From a program implementation standpoint, the outcome for program growth was the number of therapists fully trained to evaluate and treat these patients, as well as the number of outpatient clinics trained and ready to offer paired VNS sessions.

### Program implementation

Initial implementation of the paired VNS program in the AdventHealth outpatient therapy clinics began at the 3 highest-volume clinics out of the 10 clinics offering neurologic therapy services. Rather than attempt to educate all OTs, clinic supervisors, and front desk staff at all 10 clinics at once, it was decided to ramp up the program slowly to ensure smooth processes with the goal of expanding to all clinics within 1 year.

To prepare all of the neuro-specialized OTs across clinics for evaluations that require completion of the FMA-UE test, standardized training was conducted virtually, occurring 1 time per month across 3 months. Because this was not an outcome measure regularly used, it became apparent early on that building competence and confidence with the assessment would be needed for the success of the program. To monitor the program’s growth, we established criteria for OTs to be considered fully trained in providing paired VNS sessions, which include completing 1 candidacy evaluation and at least 5 paired VNS sessions.

AdventHealth Sports Medicine & Rehabilitation’s therapy sessions are typically scheduled for 45 to 60 minutes, so fitting in 90-minute sessions 3 times per week was initially viewed as a barrier. To address this, scheduling strategies were developed in advance to ensure appointments could be coordinated without disrupting standard clinic operations, such as reserving 90-minute blocks on the schedule for Vivistim patients. Although the therapy sessions were longer than traditional care at 90 minutes, there was no difference in submitting for authorization, so no additional training was required from an insurance perspective. Despite the longer session length, copays were unchanged, reducing financial barriers. Because interest in Vivistim grew among patients at clinics beyond the initial 3, we collaborated with the MicroTransponder team to provide training, in tandem with internal training, to expand access to each additional clinic, 1 at a time.

## Results

**Fig 1:**
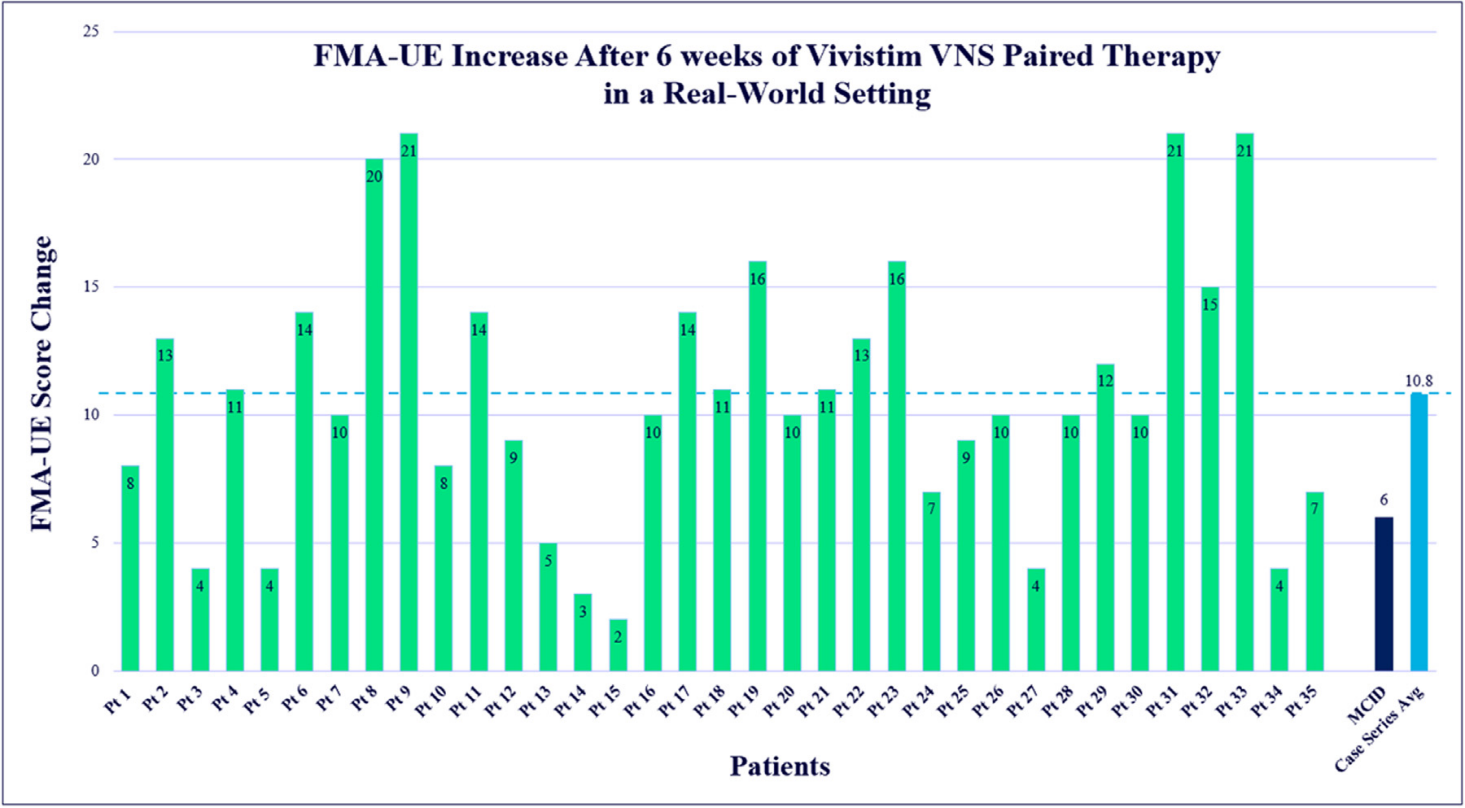
Each patient’s FMA-UE test score changes after ∼6 weeks of Vivistim Paired VNS Therapy with a range of a 2-21-point increase. The MCID score is also shown at 6.0 points, and the average from this case series at 10.8.

**Fig 2:**
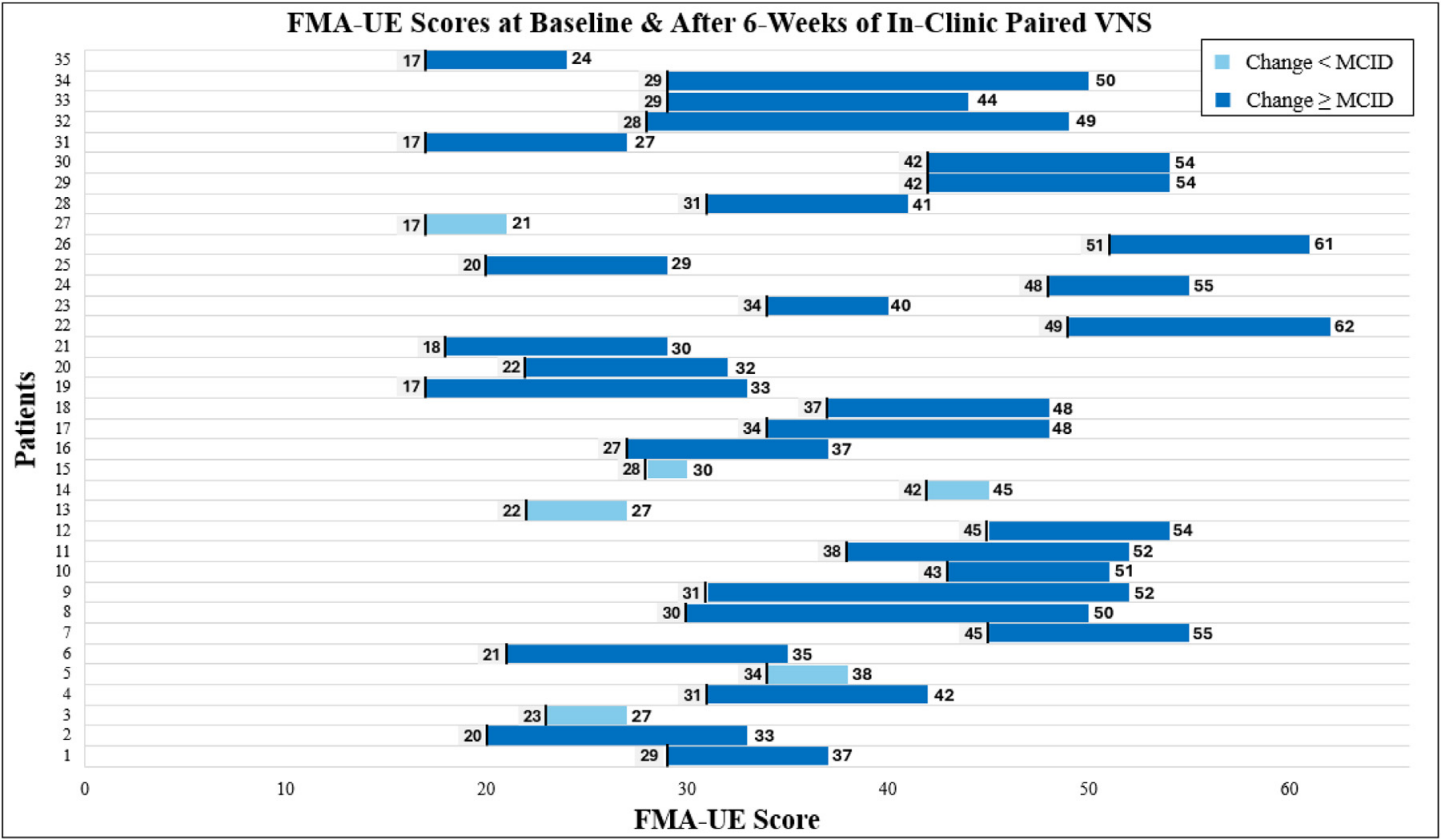
FMA-UE test scores for each patient at baseline and after ∼6 weeks of in-clinic Vivistim sessions. Bars shown in light blue depict scores below the MCID score of 6.0 points, and those in dark blue met or exceeded the MCID score.

The first 35 patients at AdventHealth to complete Vivistim Paired VNS Therapy, with both baseline and final outcome measures available, showed positive gains in both motor function and self-reported functional performance. Of the 35 patients, 28 (80%) met the MCID of at least a 6.0-point improvement on the FMA-UE test scores. The average change on the FMA-UE test score was 10.8 (± SD, 5.4) points. The FMA-UE test results are displayed in figure 1 and demonstrate the score change range from 2 to 21 points. To complement this, figure 2 presents each patient’s baseline and posttreatment FMA-UE test scores across the 6 weeks.

**Fig 3:**
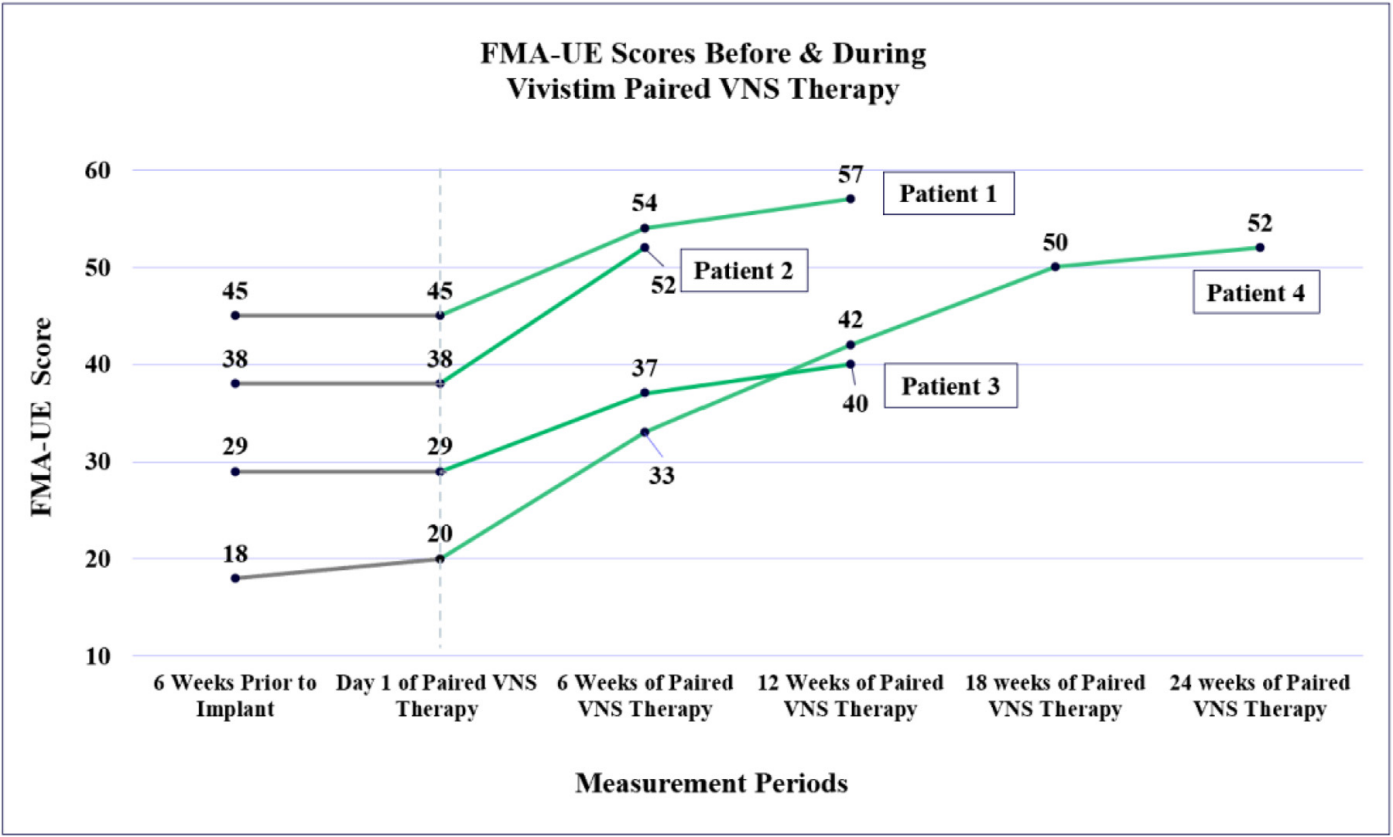
Four patients’ scores on the FMA-UE test are shown, which include scores in 6 weeks of occupational therapy before VNS implant (gray line) and each subsequent period of 6 weeks of paired VNS therapy (green line), up to 24 weeks for patient 4.

In total, 4 patients received traditional occupational therapy sessions for 45-60 minutes, 2 or 3 times per week for at least 6 weeks leading up to the start of their paired VNS sessions, allowing for a comparison of each 6-week treatment period pre- and post-VNS (fig 3). Three of these patients saw no change on the FMA-UE test scores during the pre-VNS therapy sessions, and 1 patient had a 2-point gain. After their Vivistim VNS implant procedure and 6 weeks of VNS paired therapy sessions, all 4 exceeded the FMA-UE MCID score with an average gain of 11 points (± SD, 2.5). 3 of these 4 patients continued occupational therapy sessions past the typical 6-week protocol with continued paired VNS with the SAPS software but returned to 45-60-minute sessions 2-3 times per week. The decision to continue paired VNS therapy depended on each patient’s preference, remaining goals, and insurance benefits/limitations. In the subsequent 6 weeks, the average additional increase on the FMA-UE test score for these 3 patients was 5.0 (± SD, 2.8) points, with a total average improvement in all 12 weeks of 15.0 (± SD, 5.0) points.

One patient completed 24 total weeks of Vivistim paired with VNS therapy; their FMA-UE test score improved from 20 of 66 to 52 of 66, for a 32-point improvement. Within these 24 weeks, this patient also increased his score on the BBT test from 1 block transferred to 8 blocks, and his PSFS score improved from 1.8 to 3.2 points. Functionally, by the end of 24 weeks, this patient was able to tie their shoes with both hands, open all containers, hang clothing up in their closet with the affected arm, and improve speed with household chores and meal preparation.

Additionally, 23 of 35 patients completed the BBT test. Ten patients met the minimal detectable change of at least a 5.5-point improvement in the approximate 6-week treatment period. The average improvement was 4.5 blocks/60 s (± SD, 2.9), a 210% change. Of note, 7 of the 23 patients who completed this assessment began with a score of 0 (unable to transfer any blocks), with 6 of 7 then improving to transferring at least 1 block.

Sixteen patients completed the PSFS before and after 6 weeks of Vivistim Paired VNS Therapy. The average improvement was 1.96 points (± SD, 2.21), exceeding the MCID of a 1.2-point change. The other 19 patients’ OTs either did not complete a PSFS at the end of the 6 weeks or used a different self-reported outcome measure entirely, such as the upper extremity functional index. Two patients who completed the upper extremity functional index averaged an improvement of 9.5 points, exceeding the MCID of 8.0 points.^18^

### Implementation outcomes

In addition to patient outcomes, we evaluated program implementation within the health system. Specifically, success was determined by the number of therapists and clinics trained to provide paired VNS evaluations and treatment, the feasibility of scheduling longer therapy sessions, insurance and cost considerations, and process improvements to support efficient referral and scheduling. In just over 2 years, all 10 clinics offering neurologic rehabilitation within AdventHealth achieved full training and were able to provide candidacy determination evaluations and paired VNS sessions. Of the 25 neuro-specialized OTs across these clinics, 21 (84%) became fully trained in paired VNS evaluations and treatment.

During early implementation, it became apparent that additional strategies were needed to ensure program success. For example, therapists required more support in building confidence with the FMA-UE test score assessment, so supplemental 1-on-1 and group training were added accordingly. To further support efficiency and consistency, documentation templates were developed to assist therapists in including the necessary information when evaluating a potential Vivistim candidate as well as during all treatment sessions, so more time could be spent with the patient than at the computer documenting.

We also learned early in the program that clinics typically had about 4 weeks’ notice to prepare for Vivistim therapy sessions after insurance approval because of surgical scheduling timelines and the 7-10 days of healing required postimplant. This advance notice made it unnecessary to reserve preemptive schedule blocks as we had originally planned; instead, front desk staff were instructed to schedule all 6 weeks of sessions as soon as the implant date was confirmed by the surgeon’s office or the MicroTransponder team. To further streamline coordination of referrals and therapy initiation, front desk staff received targeted training on Vivistim scheduling processes, both from the MicroTransponder team and the internal program leaders. These individuals would schedule any incoming referrals, including seeking a physician referral when necessary, and label the appointment as a “Vivistim Evaluation” to ensure the evaluating therapist was prepared and to avoid any confusion at the start of the evaluation. Collectively, these early adjustments improved scheduling efficiency, reduced delays in therapy initiation, and supported smoother integration of Vivistim into routine clinic operations.

## Discussion

The magnitude of gains in this case series exceeded those reported in the pivotal VNS-REHAB trial, suggesting that real-world implementation may yield outcomes comparable to or greater than those seen in controlled settings. By comparison, participants in the VNS-REHAB trial showed an average improvement of 5.0 points on the FMA-UE test score after 6 weeks of in-clinic therapy, whereas this case series demonstrates a greater outcome with a 10.8-point improvement. However, this series has a smaller sample size, lacks a control group, and was not blinded. This real-world example, featuring key differences from the VNS-REHAB trial, such as at-home VNS use during the initial 6-week period and a variety of rehabilitation interventions, underscores the need for further research to clarify which factors are driving the more substantial outcomes observed outside a controlled trial.

One explanation for greater gains may be early use of VNS both in and outside the clinic. To keep the VNS-REHAB trial controlled, participants only received VNS stimulations during in-clinic therapy for the first 6 weeks. Now, in the real-world setting, self-activated use of VNS can result in up to 4 hours of additional stimulation paired with functional tasks each day, though the actual frequency of use will differ from patient to patient. Self-activated VNS represents an important area for further exploration because greater frequency of use may contribute to improved outcomes. Future research should also examine whether combining other rehabilitation approaches with task-specific training, such as in this case series, is more effective than pairing VNS with task-specific training alone.

Although long-term follow-up is ongoing for this case series, early data show that in-clinic paired VNS therapy continues to be effective past the initial 6 weeks of therapy. These real-world findings support continued in-clinic paired VNS sessions after the initial 6-week period, when needed, to further improve motor recovery. Extending paired VNS therapy sessions for a period longer than 6 weeks could help reach any remaining functional goals patients have not yet achieved by the end of the initial 6-week protocol. Further studies comparing the rate of change of in-clinic therapy at intervals of 6 weeks may help occupational therapists determine therapy plans after 6 weeks because no ceiling effect was seen in response improvements after 6 weeks. If this can be demonstrated in a future controlled trial, the findings could lead to more large-scale changes for poststroke rehabilitation, such as adjustments to clinical practice guidelines and an increased amount of insurance-covered outpatient therapy visits for this population.

Implementing a Vivistim program across multiple sites may appear daunting initially, but starting small can make the process more manageable. While our program chose to train all neuro-specialized occupational therapists at each clinic so everyone would be prepared to inform patients, evaluate, and treat appropriately, others may find it more effective to designate just 1 or 2 therapists. For scheduling the therapy sessions, though there is typically at least a few weeks’ notice, placing designated blocks on the schedule specifically for Vivistim patients is another option to ensure 90-minute time slots are available when needed. One initial concern was that there would be an influx of patients at once; however, with many patients requiring prior authorization for the implant procedure, insurance approvals have been conveniently staggered, limiting overlap of more than 2 patients at the same clinic. There are many ways to go about implementing a Vivistim program, and tailoring scheduling and staffing approaches to the unique workflow of each clinic can help integrate the therapy more seamlessly.

Of the 35 patients, all but 2 completed all 18 in-clinic therapy sessions. This high completion rate likely reflects both the candidacy considerations (with many referred patients being highly motivated) and the unique commitment associated with undergoing an implant procedure, which may have positively influenced therapy adherence. Although longer than typical, the 90-minute sessions involved 1 provider, so copays matched traditional therapy, minimizing financial barriers.

While there are barriers to implementation in a health system, it is feasible to implement a successful Vivistim Paired VNS program in the real-world setting. With proper training and communication among all involved, even large health systems with multiple therapy clinics can be successful with multiple sites offering Vivistim Paired VNS Therapy. For a population typically expected to plateau in the chronic stage, Vivistim provides an option to meet functional goals when this may otherwise be out of reach. Health systems and outpatient therapy clinics should be encouraged to adopt a Vivistim Paired VNS program to expand access for stroke survivors and create meaningful improvements in their patients’ lives.

### Study limitations

This case series lacked blinding and, therefore, has some inherent bias from the occupational therapists performing outcome measure testing. Because of the nature of outpatient therapy scheduling, preoutcome and postoutcome measures were often assessed using different occupational therapists. Additionally, there was a lack of standardization in testing the BBT and PSFS, with only 16 patients completing the PSFS and 23 completing the BBT tests. While there is a small sample of patient data before the VNS implant, there is no control group in this case series for a full cohort preoutcome and postoutcome comparison. Finally, these results may have limited generalizability because they reflect the experience of a single health system with a high concentration of neuro-specialized occupational therapists, which may not represent all practice settings.

## Conclusions

This initiative assessed both the outcomes and the practical implementation of a Vivistim Paired VNS program. Participants demonstrated improvements in upper extremity motor function and functional task performance, while the program itself was integrated successfully into clinical practice. Together, these findings confirm that the stated objective to evaluate real-world outcomes and feasibility was met, supporting broader adoption of Vivistim programs within health systems.

## Disclosure

A.S. was a paid consultant with MicroTransponder, the manufacturer of Vivistim, from March 2023 to August 2025. Paid activities included meeting with health care systems to assist with program development and instructing continuing education courses focused on the therapy principles to be paired with vagus nerve stimulation in this population. As of August 2025, A.S. works full-time for MicroTransponder as the Manager of Clinical Education. The other authors have nothing to disclose.
